# Trends in the Use of Benzodiazepines, Z-Hypnotics, and Serotonergic Drugs Among US Women and Men Before and During the COVID-19 Pandemic

**DOI:** 10.1001/jamanetworkopen.2021.31012

**Published:** 2021-10-25

**Authors:** Sadaf Arefi Milani, Mukaila A. Raji, Lu Chen, Yong-Fang Kuo

**Affiliations:** 1 Department of Internal Medicine–Geriatrics and Palliative Medicine, University of Texas Medical Branch, Galveston; 2 Sealy Center on Aging, University of Texas Medical Branch, Galveston; 3 Center for Interdisciplinary Research in Women’s Health, University of Texas Medical Branch, Galveston; 4 Department of Preventive Medicine and Population Health, University of Texas Medical Branch, Galveston; 5 Office of Biostatistics, University of Texas Medical Branch, Galveston

## Abstract

**Importance:**

The ongoing COVID-19 pandemic and associated mitigation measures have disrupted access to psychiatric medications, particularly for women.

**Objective:**

To assess the sex differences in trends in the prescribing of benzodiazepines, Z-hypnotics and serotonergic (selective serotonin reuptake inhibitors [SSRIs] and serotonin and norepinephrine reuptake inhibitors [SNRIs]), which are commonly prescribed for anxiety, insomnia, and depression.

**Design, Setting, and Participants:**

This cohort study used data from Clinformatics Data Mart, one of the largest commercial health insurance databases in the US. Enrollees 18 years or older were required to have complete enrollment in a given month during our study period, January 1, 2018, to March 31, 2021, to be included for that month.

**Main Outcomes and Measures:**

Prescription of a benzodiazepine, Z-hypnotic, or SSRI or SNRI. For each month, the percentage of patients with benzodiazepine, Z-hypnotic, or SSRI or SNRI prescriptions by sex was calculated.

**Results:**

The records of 17 255 033 adults (mean [SD] age, 51.7 [19.5] years; 51.3% female) were examined in 2018, 17 340 731 adults (mean [SD] age, 52.5 [19.7] years; 51.6% female) in 2019, 16 916 910 adults (mean [SD] age, 53.7 [19.8] years; 51.9% female) in 2020, and 15 135 998 adults (mean [SD] age, 56.2 [19.8] years; 52.5% female) in 2021. Compared with men, women had a higher rate of prescriptions for all 3 drugs classes and had larger changes in prescription rates over time. Benzodiazepine prescribing decreased from January 2018 (women: 5.61%; 95% CI, 5.60%-5.63%; men: 3.03%; 95% CI, 3.02%-3.04%) to March 2021 (women: 4.91%; 95% CI, 4.90%-4.93%; men: 2.66%; 95% CI, 2.65%-2.67%), except for a slight increase in April 2020 among women. Z-hypnotic prescribing increased from January 2020 for women (1.39%; 95% CI, 1.38%-1.40%) and February 2020 for men (0.97%; 95% CI, 0.96%-0.98%) to October 2020 (women: 1.46%; 95% CI, 1.46%-1.47%; men: 1.00%; 95% CI, 0.99%-1.01%). Prescribing of SSRIs and SNRIs increased from January 2018 (women: 12.77%; 95% CI; 12.75%-12.80%; men: 5.56%; 95% CI, 5.44%-5.58%) to April 2020 for men (6.73%; 95% CI, 6.71%-6.75%) and October 2020 for women (15.18%; 95% CI, 15.16%-15.21%).

**Conclusions and Relevance:**

In this cohort study, coinciding with the COVID-19 pandemic onset was an increase in Z-hypnotic as well as SSRI and SNRI prescriptions in both men and women along with an increase in benzodiazepine prescriptions in women, findings that suggest a substantial mental health impact of COVID-19–associated mitigation measures.

## Key Points

**Question:**

What are the changes in prescribing rates in men and women of benzodiazepines, Z-hypnotics, and serotonergic drugs during the COVID-19 pandemic (2020 and 2021) compared with prior years (2018 and 2019)?

**Findings:**

This cohort study of US adults (15.1 million to 17.3 million depending on the year studied) found an increase in Z-hypnotic and serotonergic drug prescriptions in both men and women along with an increase in benzodiazepine prescriptions in women at the start of the COVID-19 pandemic.

**Meaning:**

These findings suggest a substantial association of COVID-19–associated social isolation, stay-at-home orders, and other COVID-related mitigation measures with mental health issues, especially among women.

## Teaser

This cohort study examines trends in the prescribing of benzodiazepines, serotonergic drugs, and Z-hypnotics among US adults during the COVID-19 pandemic.

## Introduction

SARS-CoV-2 has been rapidly circulating worldwide since December 2019, causing COVID-19. On March 11, 2020, the World Health Organization declared the COVID-19 outbreak a pandemic.^1^ On March 13, 2020, the US declared a national emergency, and cities began issuing shelter-in-place orders, school closures, and bans of large gatherings.^2^ The ongoing COVID-19 pandemic impact extends beyond short-term morbidity and mortality to myriad physical, cognitive, and mental health disability and morbidity effects. In particular, lockdown and shelter-in-place orders and the consequent social isolation, job loss, and income loss have been linked to a substantial increase in mental health conditions in the face of restricted access to primary mental health care and treatment.^3^

The mental health impact of the COVID-19 pandemic and the mitigation measures to stop disease spread have disproportionately affected women, not just COVID-19 survivors,^4^ but also their families, caregivers, and the general public.^5,6,7,8,9,10,11,12^ An unintended consequence of the lockdown and other isolating practices that were adopted as a result of the pandemic, as well as anxiety attributable to fear of being infected with COVID-19 and unknowns related to COVID-19, was an increase in isolation and loneliness, use of alcohol, and suicidal ideation.^8,13,14,15,16^ From August 2020 to February 2021, the percentage of adults in the US with symptoms of anxiety or depressive disorders increased from 36.4% to 41.5%.^17^ Among men, this increase was from 31.8% to 38.0%, whereas for women, the percentage of those with symptoms increased from 40.7% to 44.8%.^17^ In addition, many adults have reported difficulty sleeping,^10,18^ most of them women.^18^

Several factors might explain why women are disproportionately affected by COVID-19–related mitigation measures.^19,20,21^ First, women have higher rates of mental health conditions, such as depression and anxiety, compared with men.^22^ The ongoing COVID-19 pandemic has had an unequal effect on women, potentially exacerbating this disparity.^11^ Second, more than 75% of health care workers are women, with many of these women in positions that require prolonged close contact with patients.^23^ Third, compared with men, women are more likely to participate in the workforce that is paid outside formal channels, which lacks the legal protection that requires COVID-19 mitigation measures (eg, social distancing and testing).^24^

Fourth, most caregivers in the US are women, who experience the large toll that caregiving exacts on their well-being, including its social, emotional, and physical aspects.^25^ COVID-19 is exacerbating caregiving responsibilities that existed among women before the pandemic because, with the closures of schools and childcare centers, the burden of childcare falls disproportionately on women.^26^ Even in dual-career households with couples working from home, the burden of childcare often falls on the woman, and many women are prioritizing their household roles over their professional ones.^21^ Finally, women have been disproportionately affected by joblessness throughout the pandemic.^27^ It is thus likely that the substantial increase in caregiving demand and stress placed on women throughout the pandemic will affect women’s mental health and use of psychiatric medications. However, most health care resources have been devoted to the medical and nonpsychiatric aspects of COVID-19 care, with fewer resources allocated to address the increasing need of psychotherapy and medications (and other treatments) for mental health conditions, such as depression, anxiety, and insomnia.^15,16,17,18^ It is thus important to know the extent to which the COVID-19–related mitigation measures are associated with the patterns of prescribing of psychiatric medications commonly used to treat these mental health conditions and the association of sex with the type of psychiatric medications prescribed. Such knowledge is a key step in reducing the adverse effects (eg, suicide, substance use disorder, and gender-based violence) of untreated or undertreated mental health conditions.

This study analyzes trends in the prescribing of 3 classes of drugs: benzodiazepines drugs (antianxiety and anti-insomnia), serotonergic drugs (antianxiety and antidepressants: selective serotonin reuptake inhibitors [SSRIs] and serotonin and norepinephrine reuptake inhibitors [SNRIs]), and Z-hypnotics (nonbenzodiazepine drugs [eg, zolpidem, zaleplon, and eszopiclone] for insomnia). Although there are some pharmacodynamic and pharmacokinetic differences among medications in a particular drug class (eg, benzodiazepine drug class [diazepam vs alprazolam] and serotonergic drug class [paroxetine vs fluoxetine vs duloxetine]), we examine the psychiatric medications in each class together based on the general mechanisms of actions that underlie their indications for anxiety, depression, and/or insomnia. We are interested in how the trends in prescribing of these psychiatric medications differed by sex. We examine these medications as a proxy for mental health outcomes, which allows us to examine the complexity of the potential effects of the COVID-19–related isolation and other mitigation measures on the trajectory of mental health conditions before and during the COVID-19 pandemic. We hypothesized that the start of the pandemic and the stay-at-home order issued in March 2020 were associated with an increase in the prescribing of benzodiazepines, Z-hypnotics, and serotonergic drugs. We also hypothesized that this increase was greater for women than for men.

## Methods

### Data Collection

We conducted a cohort study using data from Clinformatics Data Mart, one of the largest commercial health insurance databases in the US. We used deidentified data from the member file to identify qualified enrollees for each month and the pharmacy file to identify the prescribed medication. Enrollees 18 years or older were required to have complete enrollment at a given month during our study period, January 1, 2018, to March 31, 2021, to be included in the denominator for that month. Informed consent was waived by the institutional review board of the University of Texas Medical Branch at Galveston because data were deidentified before our access. This study followed the Strengthening the Reporting of Observational Studies in Epidemiology (STROBE) reporting guideline for cohort studies and was approved by the institutional review board of the University of Texas Medical Branch at Galveston.

### Study Outcomes

We used National Drug Code for each drug, extracted from the *Red Book*,^28^ to identify prescriptions of benzodiazepines, Z-hypnotics, and serotonergic drugs. For each enrollee, we accounted for the date of prescription and the days of supply to assess whether the enrollee had at least 1 day of prescription of 1 of these drugs for the numerator for the given month and drug.

### Statistical Analysis

We summarized patient characteristics for 2018, 2019, 2020, and 2021, including age and region of residence (Northeast, Midwest, South, and West) for the total sample and by sex. For each month from January 1, 2018, to March 31, 2021, we then calculated the percentage of patients with SSRI or SNRI, benzodiazepine, or Z-hypnotic prescriptions by sex. We also included metformin as a control drug to assess the general prescribing variance during our study period. These monthly rates of prescriptions were plotted for each drug by sex and age, and joinpoint regression models, with a maximum of 5 possible joinpoints, were used to assess where time trends had significant changes. A sequential application of the permutation test using 4500 possible randomly permuted data sets and a bayesian information criterion were used to determine the optimal number of joinpoints. A 2-sided *P* < .05 was considered to be statistically significant. All analyses were conducted with SAS software, version 9.4 (SAS Institute Inc) and the Joinpoint Regression Program, version 4.4.0.0 (National Cancer Institute).^29^

## Results

### Demographics

The records of 17 255 033 adults (mean [SD] age, 51.7 [19.5] years; 51.3% female) were examined in 2018, 17 340 731 adults (mean [SD] age, 52.5 [19.7] years; 51.6% female) in 2019, 16 916 910 adults (mean [SD] age, 53.7 [19.8] years; 51.9% female) in 2020, and 15 135 998 adults (mean [SD] age, 56.2 [19.8] years; 52.5% female) in 2021 (Table 1). The median (IQR) age increased from 52.0 years (35.0-68.0 years) in 2018 to 59.0 years (39.0-72.0 years) in 2021 (women: 54.0 to 62.0 years; men: 50.0 to 57.0 years). The distribution of participants living in each region did not change between 2018 and 2021. Data on race, ethnicity, and socioeconomic status were not available.

**Table 1.** Characteristics of Enrollees 18 Years and Older in the US, 2018-2021^a^

Characteristic	Year			
	2018 (n = 17 255 033 [8 846 497 female and 8 408 536 male])	2019 (n = 17 340 731 [8 948 495 female and 8 392 236 male])	2020 (n = 16 916 910 [8 783 927 female and 8 132 983 male])	2021 (n = 15 135 998 [7 939 181 female and 7 196 817 male])
**Total**				
Age, y				
Mean (SD)	51.7 (19.5)	52.5 (19.7)	53.7 (19.8)	56.2 (19.8)
Median (IQR)	52.0 (35.0-68.0)	53.0 (35.0-69.0)	55.0 (36.0-70.0)	59.0 (39.0-72.0)
Region				
Northeast	1 788 695 (10.7)	1 842 159 (10.9)	1 823 130 (11.1)	1 659 741 (11.2)
Midwest	3 822 866 (22.8)	3 839 035 (22.7)	3 836 297 (23.3)	3 500 192 (23.6)
South	7 308 215 (43.5)	7 341 042 (43.5)	7 044 844 (42.7)	6 206 420 (41.9)
West	3 867 511 (23.0)	3 859 491 (22.9)	3 792 291 (23.0)	3 437 509 (23.2)
**Female**				
Age, y				
Mean (SD)	53.1 (19.8)	53.9 (20.0)	55.2 (20.0)	57.6 (19.9)
Median (IQR)	54.0 (36.0-70.0)	55.0 (36.0-70.0)	57.0 (38.0-71.0)	62.0 (41.0-73.0)
Region				
Northeast	933 563 (10.8)	962 985 (11.0)	957 662 (11.1)	875 002 (11.2)
Midwest	1 948 698 (22.5)	1 969 337 (22.5)	1 979 151 (23.0)	1 818 739 (23.3)
South	3 782 107 (43.7)	3 825 449 (43.7)	3 695 201 (42.9)	3 293 078 (42.2)
West	1 995 690 (23.0)	2 001 872 (22.9)	1 980 486 (23.0)	1 810 926 (23.2)
**Male**				
Age, y				
Mean (SD)	50.2 (19.1)	51.0 (19.3)	52.2 (19.4)	54.7 (19.6)
Median (IQR)	50.0 (34.0-66.0)	51.0 (34.0-67.0)	53.0 (35.0-68.0)	57.0 (38.0-71.0)
Region				
Northeast	854 977 (10.5)	879 049 (10.8)	865 286 (11.0)	784 560 (11.2)
Midwest	1 873 876 (23.1)	1 869 488 (23.0)	1 856 870 (23.6)	1 681 142 (24.0)
South	3 525 225 (43.4)	3 514 882 (43.3)	3 348 869 (42.5)	2 912 601 (41.6)
West	1 871 554 (23.0)	1 857 362 (22.9)	1 811 372 (23.0)	1 626 133 (23.2)

^a^ Data are presented as number (percentage) of patients unless otherwise indicated. The total number of patients in all years was 7 995 773.

### Benzodiazepines

**Figure 1.**
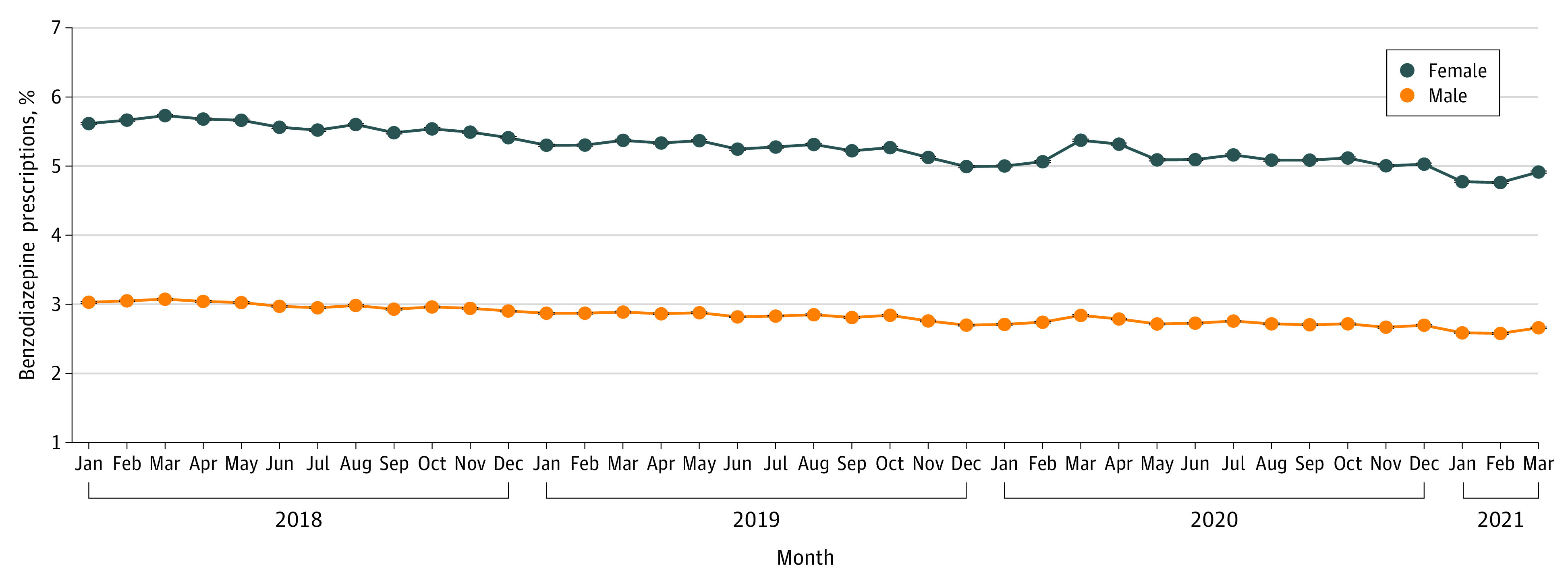
Monthly Rates of Benzodiazepine Prescriptions Among Adults 18 Years or Older in the US, 2018-2021, by Sex

Figure 1 displays the monthly benzodiazepine prescribing rates by sex from January 2018 to March 2021. For women, the prescribing rates decreased from 5.61% (95% CI, 5.60%-5.63%) in January 2018 to 5.00% (95% CI, 4.98%-5.02%) in January 2020, increased to 5.32% (95% CI, 5.30%-5.33%) in April 2020, and decreased to 4.91% (95% CI, 4.90%-4.93%) in March 2021. For men, the prescribing rates decreased from 3.03% (95% CI, 3.02%-3.04%) in January 2018 to 2.66% (95% CI, 2.65%-2.67%) in March 2021. For women, the prescription rates of benzodiazepines decreased 0.03% monthly from January 2018 to January 2020, increased 0.06% monthly from January 2020 to April 2020, and decreased 0.04% monthly from April 2020 to March 2021. For men, the joinpoint analysis found no significant changes in the slope of rates of benzodiazepine prescription from January 2018 to March 2021 (slope, −1.1 × 10^−2^; *P* < .001) (Table 2). The monthly decrease in benzodiazepine prescription rate from January 2018 to March 2021 for men was 0.01%. The monthly decrease in the benzodiazepine prescription rate from January 2018 to March 2021 was 0.01% for those 18 to 49 years of age and 0.03% for those 50 to 64 years of age. For those 65 years and older, the monthly decrease in prescribing rate from January 2018 to December 2019 was 0.03%, followed by a monthly increase of 0.04% from December 2019 to March 2020, followed by a monthly decrease of 0.05% from March 2020 to March 2021 (eFigure 1 and eTable in the Supplement).

**Table 2.** Joinpoints in Trends of Monthly Prescribing of Benzodiazepines, Z-Hypnotics, and SSRIs and SNRIs Among Adults 18 Years or Older in the US, January 2018 to March 2021

Study period	Slope^a^	*P* value
**Benzodiazepines**		
Total		
Jan 2018-Jan 2020	−2.0 × 10^−2^	<.001
Jan 2020-Apr 2020	3.9 × 10^−2^	.60
Apr 2020-Mar 2021	−2.8 × 10^−2^	<.001
Female		
Jan 2018-Jan 2020	−2.7 × 10^−2^	<.001
Jan 2020-Apr 2020	6.0 × 10^−2^	.56
Apr 2020-Mar 2021	−4.0 × 10^−2^	<.001
Male		
Jan 2018-Mar 2021	−1.1 × 10^−2^	<.001
**Z-hypnotics**		
Total		
Jan 2018-Mar 2018	5.4 × 10^−2^	.002
Mar 2018-Jan 2020	−3.7 × 10^−3^	<.001
Jan 2020-Oct 2020	4.6 × 10^−3^	.01
Oct 2020-Mar 2021	−6.7 × 10^−3^	.07
Female		
Jan 2018-Mar 2018	6.7 × 10^−2^	.001
Mar 2018-Jan 2020	−4.6 × 10^−3^	<.001
Jan 2020-Oct 2020	5.7 × 10^−3^	.01
Oct 2020-Mar 2021	−9.1 × 10^−3^	.04
Male		
Jan 2018-Mar 2018	4.0 × 10^−2^	.005
Mar 2018-Feb 2020	−3.0 × 10^−3^	<.001
Feb 2020-Oct 2020	3.7 × 10^−3^	.04
Oct 2020-Mar 2021	−5.3 × 10^−3^	.13
**SSRIs and SNRIs**		
Total		
Jan 2018-Sep 2020	5.3 × 10^−2^	<.001
Sep 2020-Mar 2021	−2.4 × 10^−2^	.53
Female		
Jan 2018-Oct 2020	6.5 × 10^−2^	<.001
Oct 2020-Mar 2021	−5.4 × 10^−2^	.44
Male		
Jan 2018-Jan 2020	3.1 × 10^−2^	<.001
Jan 2020-Apr 2020	1.1 × 10^−1^	.30
Apr 2020-Mar 2021	−9.5 × 10^−3^	.20

Abbreviations: SNRI, serotonin and norepinephrine reuptake inhibitor; SSRI, selective serotonin reuptake inhibitor.

^a^ Slope is expressed as change in prescription rate per month; for example, −2.0 × 10^−2^ means 0.02% decrease in benzodiazepine prescription rate per month.

### Z-Hypnotics

**Figure 2.**
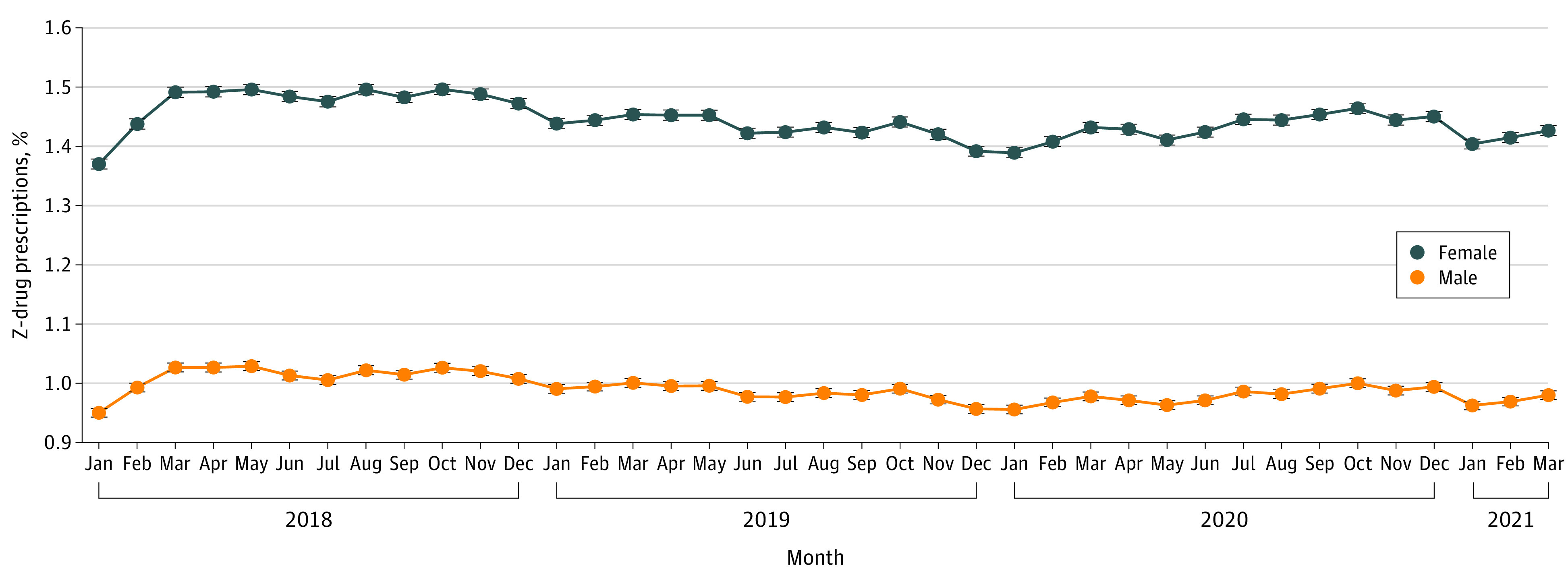
Monthly Rates of Z-Hypnotic Prescriptions Among Adults 18 Years or Older in the US, 2018-2021, by Sex Z-hypnotics are nonbenzodiazepine drugs, such as zolpidem, zaleplon, and eszopiclone.

For women, prescribing rates increased from 1.37% (95% CI, 1.36%-1.38%) in January 2018 to 1.49% (95% CI, 1.48%-1.50%) in March 2018, then decreased to 1.39% (95% CI, 1.38%-1.40%) in January 2020, increased to 1.46% (95% CI, 1.46%-1.47%) in October 2020, and decreased to 1.43% (95% CI, 1.42%-1.44%) in March 2021 (Figure 2). For men, these rates increased from 0.95% (95% CI, 0.94%-0.96%) in January 2018 to 1.03% (95% CI, 1.02%-1.03%) in March 2018, then decreased to 0.97% (95% CI, 0.96%-0.98%) in February 2020, increased to 1.00% (95% CI, 0.99%-1.01%) in October 2020, and decreased to 0.98% (95% CI, 0.97%-0.99%) in March 2021.

From January 2018 to March 2018, the overall monthly increase in prescribing rate was 0.07% for women and 0.04% for men (Table 2). For women, from March 2018 to January 2020, the monthly decrease in prescribing rates was 0.005%, followed by a monthly increase of 0.006% from January 2020 to October 2020. For men, from March 2018 to February 2020, the monthly decrease in prescribing rates was 0.003%, followed by a monthly increase of 0.004% from January 2020 to October 2020. From October 2020 to March 2021, the monthly decrease in prescribing rates was 0.009% for women and 0.005% for men. For those 18 to 49 and 50 to 64 years of age, there was no statistically significant change in the monthly prescription rates of Z-hypnotic prescriptions from January 2018 to January 2020. From January 2020 to October 2020, the monthly increase in prescribing rates was 0.006% for those 18 to 49 years of age and 0.01% for those 50 to 64 years of age, followed by a decrease to 0.01% for those 18 to 49 years of age and 0.03% for those 50 to 64 years of age from October 2020 to March 2021. For those 65 years and older, the monthly increase in prescribing rates was 0.1% from January 2018 to March 2018, followed by a monthly increase of 0.008% from March 2018 to March 2019. After March 2019, there were no statistically significant changes in the slope of monthly prescribing rates for those 65 years and older (eFigure 2 and eTable in the Supplement).

### SSRIs and SNRIs

**Figure 3.**
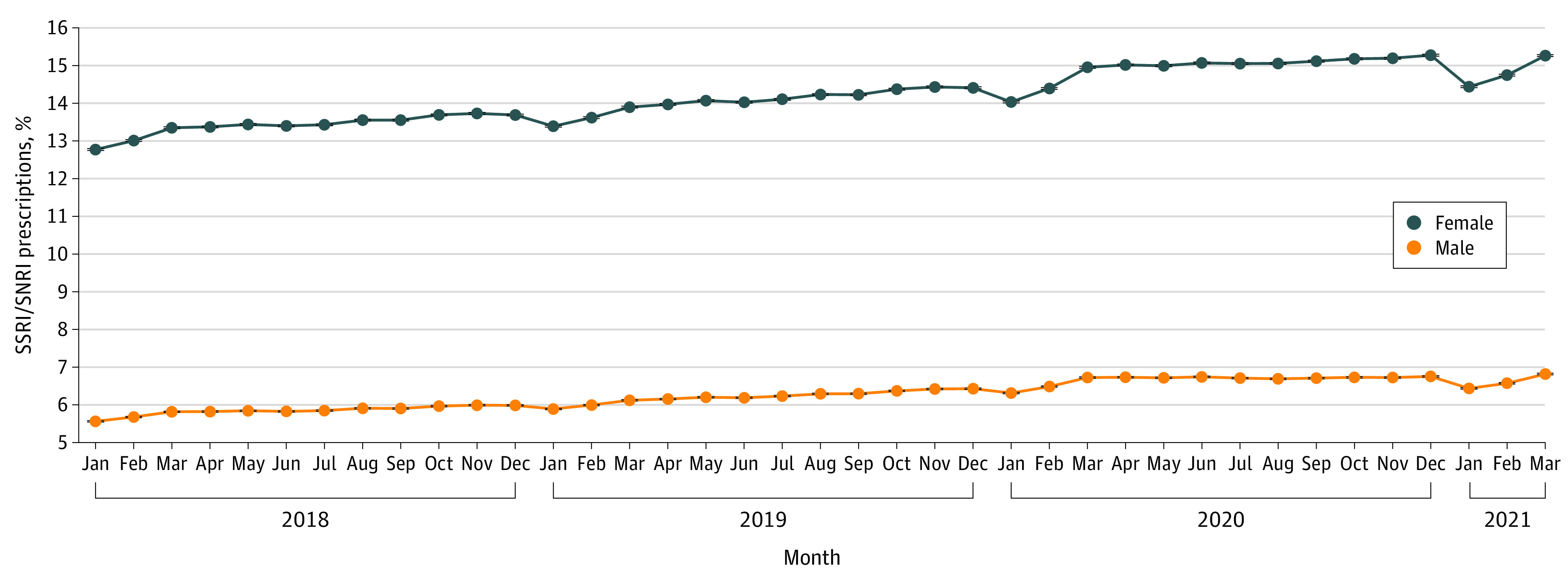
Monthly Rates of Serotonergic Drug Prescriptions Among Adults 18 Years or Older in the US, 2018-2021, by Sex Serotonergic drugs are selective serotonin reuptake inhibitors and serotonin and norepinephrine reuptake inhibitors.

For women, SSRI and SNRI prescribing rates increased from 12.77% (95% CI, 12.75%-12.80%) in January 2018 to 15.18% (95% CI, 15.16%-15.21%) in October 2020, followed by a decrease to 14.75% (95% CI, 14.72%-14.77%) in February 2021 (Figure 3). For men, SSRI and SNRI prescribing rates increased from 5.56% (95% CI, 5.44%-5.58%) in January 2018 to 6.31% (95% CI, 6.29%-6.33%) in January 2020, then increased to 6.73% (95% CI, 6.71%-6.75%) in April 2020, followed by a decrease to 6.57% (95% CI, 6.55%-6.59%) in February 2021.

For women, the monthly increase in prescription rates of SSRIs and SNRIs was 0.07% from January 2018 to October 2020, followed by a 0.05% decrease in rates from October 2020 to March 2021. For men, rates increased 0.03% monthly from January 2018 to January 2020 and 0.1% monthly from January 2020 to April 2020, followed by a 0.01% monthly decrease from April 2020 to March 2021. For those 18 to 49 years of age, the monthly increase in the SSRI and SNRI prescription rates from January 2018 to March 2021 was 0.04%. For those 50 to 64 years of age, the monthly increase in the SSRI and SNRI prescription rates was 0.05% from January 2018 to October 2020. After October 2020, there were no statistically significant changes in the slope of monthly prescribing rates for those 50 to 64 years of age. For those 65 years and older, the monthly increase in the SSRI and SNRI prescription rates was 0.05% from January 2018 to August 2020, followed by a monthly decrease of 0.09% from August 2020 to March 2021 (eFigure 3 and eTable in the Supplement).

### Metformin

For women, prescribing rates increased from 5.71% (95% CI, 5.69%-5.72%) in January 2018 to 6.69% (95% CI, 6.67%-6.71%) in June 2020, then decreased to 6.63% (95% CI, 6.67%-6.71%) in March 2021 (eTable and eFigure 4 in the Supplement). For men, prescribing rates increased from 6.13% (95% CI, 6.11%-6.15%) in January 2018 to 7.34% (95% CI, 7.32%-7.36%) in May 2020, then decreased to 7.26% (95% CI, 7.24%-7.28%) in February 2021. eFigure 5 and eTable in the Supplement display aged-stratified monthly prescriptions of metformin and joinpoints in trends of prescribing.

## Discussion

This cohort study found that coinciding with the COVID-19 pandemic onset was an increase in Z-hypnotic as well as SSRI and SNRI prescriptions in both men and women, along with an increase in benzodiazepine prescriptions in women, findings that underscore the substantial mental health impact of COVID-19–associated social isolation, stay-at-home orders, and other mitigation measures. The increase in Z-hypnotic as well as SSRI and SNRI prescriptions persisted throughout the first and second waves, whereas the increase in benzodiazepine prescriptions among women reflects the first wave.

The increase in prescribing of Z-hypnotics as well as SSRIs and SNRIs, medications commonly used for insomnia, anxiety, and depressive disorders, is consistent with recent findings from Holland et al,^15^ who found that the rates of emergency department (ED) visits for mental health conditions was higher from March 2020 to October 2020 compared with the same period in the previous year. In addition, our findings of significant increases in psychiatric medication prescribing are consistent with the increased 30.9% prevalence of symptoms of anxiety disorder or depressive disorder reported by Czeisler et al^16^ in their national surveys of Americans 18 years or older in June 2020. A follow-up survey by Czeisler et al,^30^ conducted in September 2020, found a slight increase to 33.0% in the prevalence of anxiety or depression symptoms.

The increased prescribing of Z-hypnotics is of concern, especially among older adults, given the association of prolonged Z-hypnotic use with increased risk of falls or fractures and ED visits.^31,32^ In their analysis of ED visits for adverse drug events, Hampton et al^32^ found that zolpidem tartrate, the most commonly used Z-hypnotic, accounted for 11.5% of all ED visits for adverse drugs events associated with adult psychiatric medications more than any other psychiatric medication. In our analysis, women had higher rates of Z-hypnotic prescriptions compared with men. Previous research^33^ has found that women are more often prescribed zolpidem tartrate. However, we also found that women experienced a faster increase in monthly prescription rates of Z-hypnotics compared with men. This difference may reflect the unequal mental health burden and challenges of the COVID-19 pandemic that are unique to women. In our age-stratified analyses, we found that Z-hypnotic prescriptions increased from November 2019 to March 2021 for adults 65 years and older. This finding is especially concerning given the increased risk of adverse outcomes.^31,32^

The prescribing rates for benzodiazepines steadily decreased from January 2018 to March 2021, except for an increase from January 2020 to April 2020 for women, which may reflect the ongoing multilevel efforts to reduce benzodiazepine prescription. In 2016, the US Food and Drug Administration(FDA) issued its highest warning—a black box warning—that practitioners must not coprescribe opioids and benzodiazepines.^34^ In September 2020, the FDA updated the warnings to be implemented for all benzodiazepines and warned of the risk of abuse, misuse, addiction, dependence, and withdrawal.^35^ In addition, as part of the Choosing Wisely Campaign, the American Geriatrics Society identified 10 treatments with risks that may outweigh the benefits among older adults.^36^ One of the recommendations from this campaign was to not use benzodiazepines or other sedative-hypnotics as a first-choice drug for insomnia, agitation, or delirium among older adults.^36^

Compared with men, women had a higher rate of prescriptions for all 3 drugs classes and had larger changes in prescription rates over time, suggesting that the sex disparity in mental health has been exacerbated by the pandemic. Government and public health responses often leave out aspects of the COVID-19 pandemic that are unique to women.^19^ In addition, with the increased sex disparity in the burden of caregiving, the pandemic may provide an opportunity to make structural changes in economic and societal policies that value caregiving.^20^ Sex should be a priority and a key variable in all aspects of research on the COVID-19 pandemic.^19^ In addition to including sex as a research priority, interventions should also be implemented to address sex disparities in the psychological consequences of the COVID-19 pandemic.^19^

It is unclear why there was a slight decrease in the rate of monthly Z-hypnotic as well as SSRI and SNRI prescriptions after October 2020 and throughout the third wave. This finding may suggest that COVID-19–related mitigation measures have had a greater immediate effect on the rate of psychiatric medication prescriptions rather than a longer-term impact or may reflect the relaxing of mitigation measures. In addition, individuals may have gotten used to the daily stress of the pandemic. The immediate impact of lockdown also may explain the brief increase in benzodiazepine prescriptions among women. The examination of trends in metformin prescribing also suggest that the prescribing changes observed in the 3 classes of mental health drugs we studied are associated with variance in mental health drug prescribing rather than variance in general prescribing during the study period.

### Limitations

This study has some limitations. First, our sample, enrollees 18 years and older of commercial insurance programs, is not representative of the US population. Second, prescriptions reflect what was prescribed not use and treatment adherence. Third, information on race and ethnicity as well as socioeconomic status for enrollees was not available. Fourth, we examined only individuals with private insurance, excluding those who may have lost their job as a result of the pandemic. This approach may have underestimated the extent of the problem because these individuals may be the least stressed of the population affected by COVID because they are likely working and perhaps with greater opportunity for virtual work than the general population with nonprivate health insurance. We also acknowledge that some pharmacodynamic and kinetic differences among medications in a particular drug class (serotonergic drug class [paroxetine vs fluoxetine vs duloxetine]) might yield different trends if individual psychiatric medication in a class is analyzed separately—this is an area for future study, especially as new psychiatric medications are approved by the FDA. Fifth, we examined biological sex; however, we acknowledge the fluidity, multiplicity, and nonbinary nature of gender.

## Conclusions

In this cohort study, the increase in benzodiazepine prescriptions from January 2020 to April 2020 among women, during the first COVID-19 wave, and the increase in Z-hypnotic as well as SSRI and SNRI prescribing for most of 2020, throughout the first and second COVID-19 waves, may reflect the mental health impact of the COVID-19 pandemic. These findings suggest that mitigation measures, including social isolation and stay-at-home orders, might have negatively affected mental health, particularly among women. As data accumulate, examination of long-term consequences of these prescribing trends and how they relate to mental health outcomes, such as suicide and ED visits, is warranted.

## Notes

Supplement. **eTable.** Joinpoints in Trends of Monthly Prescribing of Benzodiazepines, Z-Hypnotics, SSRI/SNRI, and Metformin Among Adults Aged ≥18 in the US From January 2018 to March 2021 **eFigure 1.** Monthly Rates of Benzodiazepine (Benzo) Prescriptions Among Adults Aged ≥18 in the US, 2018-2021, by Age **eFigure 2.** Monthly Rates of Z-Hypnotic Prescriptions Among Adults Aged ≥18 in the US, 2018-2021, by Age **eFigure 3.** Monthly Rates of SSRI/SNRI Prescriptions Among Adults Aged ≥18 in the US, 2018-2021, by Age **eFigure 4.** Monthly Rates of Metformin Prescriptions Among Adults Aged ≥18 in the US, 2018-2021, by Sex **eFigure 5.** Monthly Rates of Metformin Prescriptions Among Adults Aged ≥18 in the US, 2018-2021, by Age
