# Supplementary material for: Trends in the Use of Benzodiazepines, Z-Hypnotics, and Serotonergic Drugs Among US Women and Men Before and During the COVID-19 Pandemic
Source: JAMA Netw Open. 2021 Oct 25;4(10):e2131012. doi: 10.1001/jamanetworkopen.2021.31012 (PMC8546497; doi:10.1001/jamanetworkopen.2021.31012)
Supplement: Supplement. — eTable. Joinpoints in Trends of Monthly Prescribing of Benzodiazepines, Z-Hypnotics, SSRI/SNRI, and Metformin Among Adults Aged ≥18 in the US From January 2018 to March 2021 eFigure 1. Monthly Rates of Benzodiazepine (Benzo) Prescriptions Among Adults Aged ≥18 in the US, 2018-2021, by Age eFigure 2. Monthly Rates of Z-Hypnotic Prescriptions Among Adults Aged ≥18 in the US, 2018-2021, by Age eFigure 3. Monthly Rates of SSRI/SNRI Prescriptions Among Adults Aged ≥18 in the US, 2018-2021, by Age eFigure 4. Monthly Rates of Metformin Prescriptions Among Adults Aged ≥18 in the US, 2018-2021, by Sex eFigure 5. Monthly Rates of Metformin Prescriptions Among Adults Aged ≥18 in the US, 2018-2021, by Age [file jamanetwopen-e2131012-s001.pdf]

Supplementary Online Content

Milani SA, Raji MA, Chen L, Kuo YF. Trends in the use of benzodiazepines, Z-hypnotics, and serotonergic drugs among US women and men before and during the COVID-19 pandemic. *JAMA Netw Open*. 2021;4(10):e2131012. doi:10.1001/jamanetworkopen.2021.31012

eTable. Joinpoints in Trends of Monthly Prescribing of Benzodiazepines, Z-Hypnotics, SSRI/SNRI, and Metformin Among Adults Aged ≥18 in the US From January 2018 to March 2021
eFigure 1. Monthly Rates of Benzodiazepine (Benzo) Prescriptions Among Adults Aged ≥18 in the US, 2018-2021, by Age
eFigure 2. Monthly Rates of Z-Hypnotic Prescriptions Among Adults Aged ≥18 in the US, 2018-2021, by Age
eFigure 3. Monthly Rates of SSRI/SNRI Prescriptions Among Adults Aged ≥18 in the US, 2018-2021, by Age
eFigure 4. Monthly Rates of Metformin Prescriptions Among Adults Aged ≥18 in the US, 2018-2021, by Sex
eFigure 5. Monthly Rates of Metformin Prescriptions Among Adults Aged ≥18 in the US, 2018-2021, by Age

This supplementary material has been provided by the authors to give readers additional information about their work.

© 2021 Milani SA et al. *JAMA Network Open.*

eTable. Joinpoints in Trends of Monthly Prescribing of Benzodiazepines, Z-Hypnotics, SSRI/SNRI, and Metformin Among Adults Aged ≥18 in the US From January 2018 to March 2021

**Prescription Drug Class**	**Population**	**Time Period**	**Slope* (p-value)**
**Benzodiazepines**	18-49 y	Jan 2018 – Mar 2021	-1.0×10^{-2} * (<0.001)
	50-64 y	Jan 2018 – Mar 2021	-2.8×10^{-2} * (<0.001)
	>-65 y	Jan 2018 – Dec 2019	-3.1×10^{-2} * (<0.001)
		Dec 2019 – Mar 2020	3.7×10^{-2} (0.65)
		Mar 2020 – Mar 2021	-4.5×10^{-2} * (<0.001)
**Z-Hypnotics**	18-49 y	Jan 2018 – Nov 2018	-8.0×10^{-4} (0.52)
		Nov 2018 – Feb 2019	-2.3×10^{-2} (0.16)
		Feb 2019 - Oct 2019	1.0×10^{-4} (0.96)
		Oct 2019 – Jan 2020	-2.2×10^{-2} (0.15)
		Jan 2020 – Oct 2020	5.6×10^{-3} * (0.003)
		Oct 2020 – Mar 2021	-1.4×10^{-2} * (<0.001)
	50-64 y	Jan 2018 – Nov 2018	-3.2×10^{-3} (0.28)
		Nov 2018 – Feb 2019	-4.8×10^{-2} (0.19)
		Feb 2019 - Oct 2019	-9.6×10^{-4} (0.84)
		Oct 2019 – Jan 2020	-5.0×10^{-2} (0.17)
		Jan 2020 – Oct 2020	9.5×10^{-3} * (0.02)
		Oct 2020 – Mar 2021	-2.6×10^{-2} * (0.004)
	>=65 y	Jan 2018 – Mar 2018	1.0×10^{-1} * (<0.001)
		Mar 2018 – Mar 2019	7.9×10^{-3} * (<0.001)
		Mar 2019 – Nov 2019	-2.0×10^{-3} (0.41)
		Nov 2019 – Feb 2020	2.0×10^{-2} (0.25)
		Feb 2020 – Mar 2021	1.6×10^{-3} (0.09)
**SSRI/SNRI**	18-49 y	Jan 2018 – Mar 2021	4.4×10^{-2} * (<0.001)
	50-64 y	Jan 2018 – Oct 2020	4.5×10^{-2} * (<0.001)

© 2021 Milani SA et al. *JAMA Network Open.*

		Oct 2020 – Mar 2021	-6.4×10^{-2} (0.37)
	>=65 y	Jan 2018 – Aug 2020	4.8×10^{-2} * (<0.001)
		Aug 2020 – Mar 2021	-8.9×10^{-2} * (0.01)
**Metformin**	18-49 y	Jan 2018 – Oct 2018	1.8×10^{-2} * (0.001)
		Oct 2018 – Jan 2019	-4.6×10^{-2} (0.41)
		Jan 2019 – Oct 2019	1.9×10^{-2} * (0.005)
		Oct 2019 – Jan 2020	-5.3×10^{-2} (0.36)
		Jan 2020 – Jul 2020	3.8×10^{-2} * (0.01)
		Jul 2020 – Mar 2021	-1.5×10^{-2} * (0.03)
	50-64 y	Jan 2018 – Oct 2018	7.6×10^{-2} * (<0.001)
		Oct 2018 – Jan 2019	-1.8×10^{-1} (0.40)
		Jan 2019 – Oct 2019	6.4×10^{-2} * (0.01)
		Oct 2019 – Jan 2020	-1.9×10^{-1} (0.36)
		Jan 2020 – Jul 2020	1.8×10^{-1} * (0.01)
		Jul 2020 – Mar 2021	-5.2×10^{-2} * (0.01)
	>=65 y	Jan 2018 – Feb 2020	4.7×10^{-3} (0.40)
		Feb 2020 – Jun 2020	1.8×10^{-1} (0.20)
		Jun 2020 – Mar 2021	-9.3×10^{-2} * (<0.001)
	Total	Jan 2018 – Feb 2020	1.7×10^{-2} * (<0.001)
		Feb 2020 – Jun 2020	1.4×10^{-1} (0.06)
		Jun 2020 – Mar 2021	-2.5×10^{-2} (0.07)
	Female	Jan 2018 – Feb 2020	1.3×10^{-2} * (<0.001)
		Feb 2020 – Jun 2020	1.4×10^{-1} (0.07)
		Jun 2020 – Mar 2021	-3.2×10^{-2} * (0.02)
	Male	Jan 2018 – Feb 2020	2.2×10^{-2} * (<0.001)
		Feb 2020 – May 2020	1.9×10^{-1} (0.20)
		May 2020 – Mar 2021	-9.4×10^{-3} (0.42)

© 2021 Milani SA et al. *JAMA Network Open.*

eFigure 1. Monthly Rates of Benzodiazepine (Benzo) Prescriptions Among Adults Aged ≥18 in the US, 2018-2021, by Age

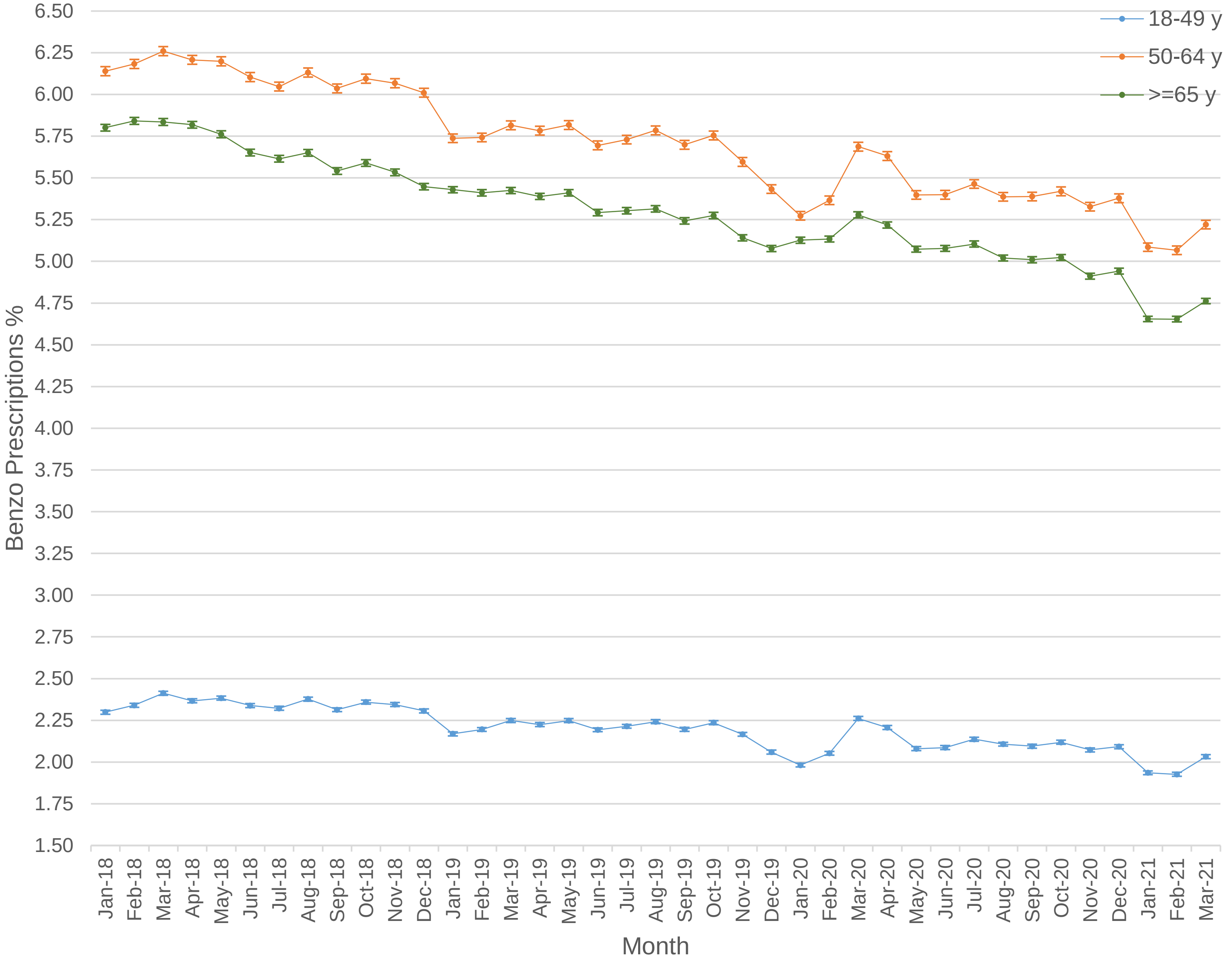

© 2021 Milani SA et al. *JAMA Network Open.*

eFigure 2. Monthly Rates of Z-Hypnotic Prescriptions Among Adults Aged ≥18 in the US, 2018-2021, by Age

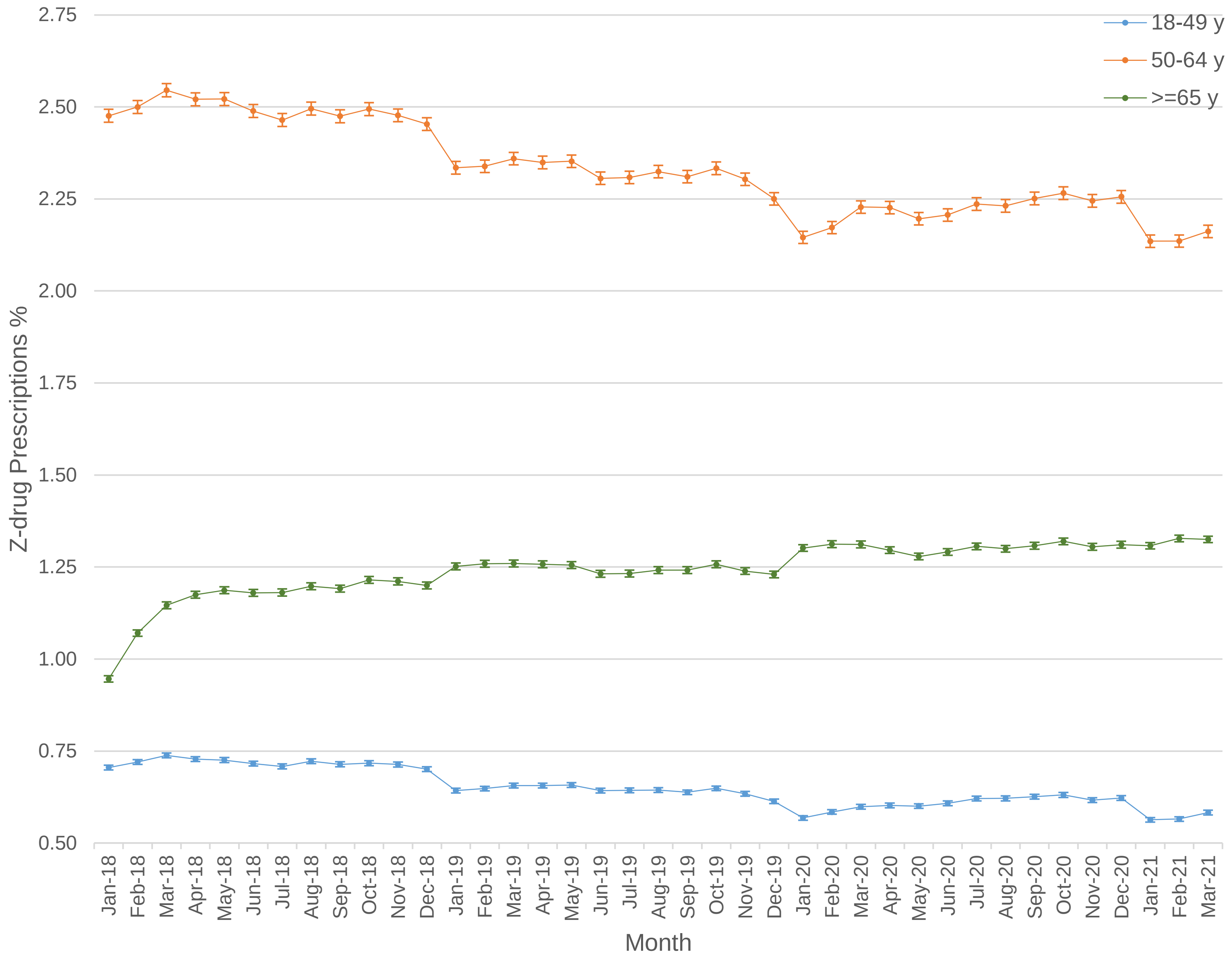

© 2021 Milani SA et al. *JAMA Network Open.*

eFigure 3. Monthly Rates of SSRI/SNRI Prescriptions Among Adults Aged ≥18 in the US, 2018-2021, by Age

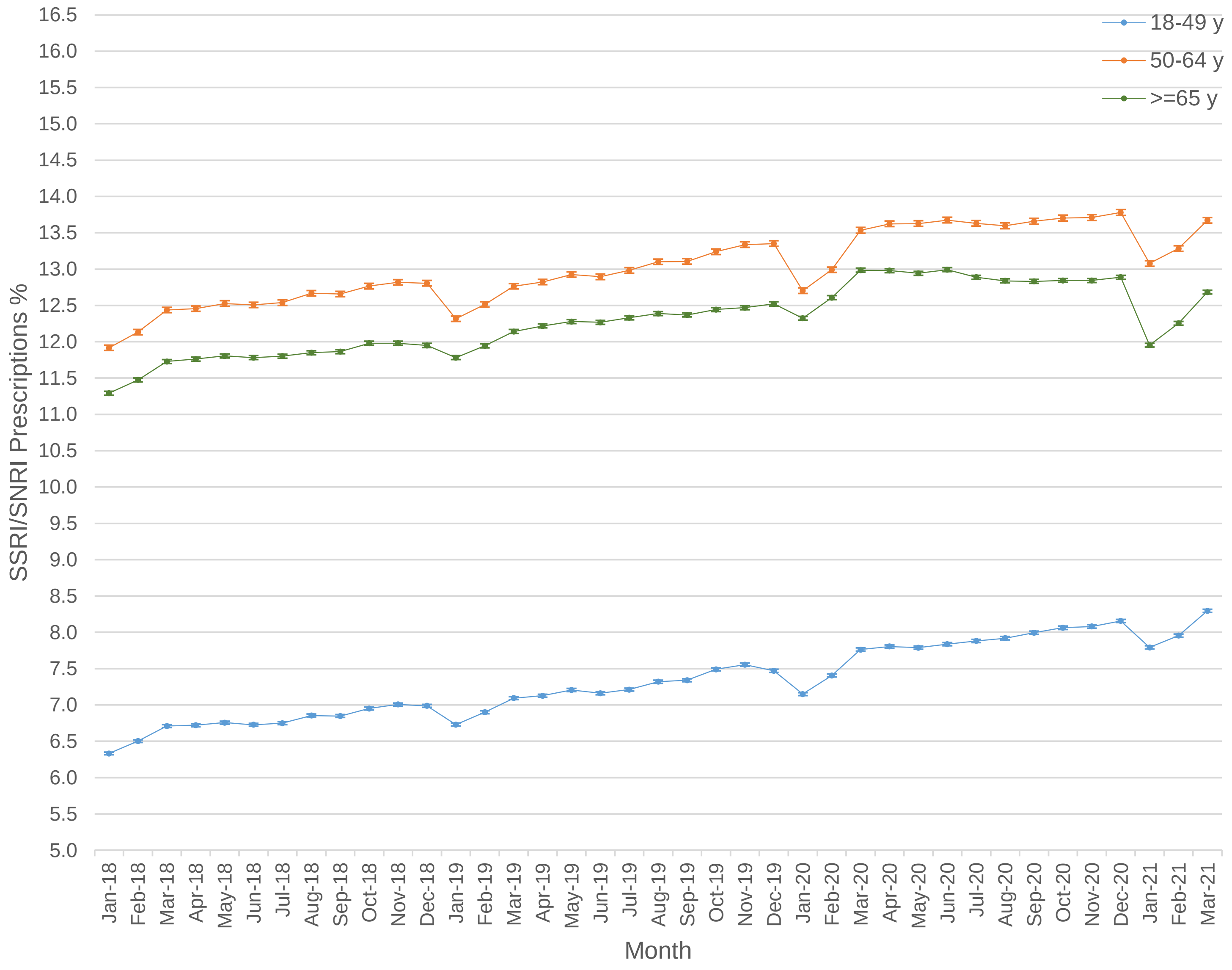

© 2021 Milani SA et al. *JAMA Network Open.*

eFigure 4. Monthly Rates of Metformin Prescriptions Among Adults Aged ≥18 in the US, 2018-2021, by Sex

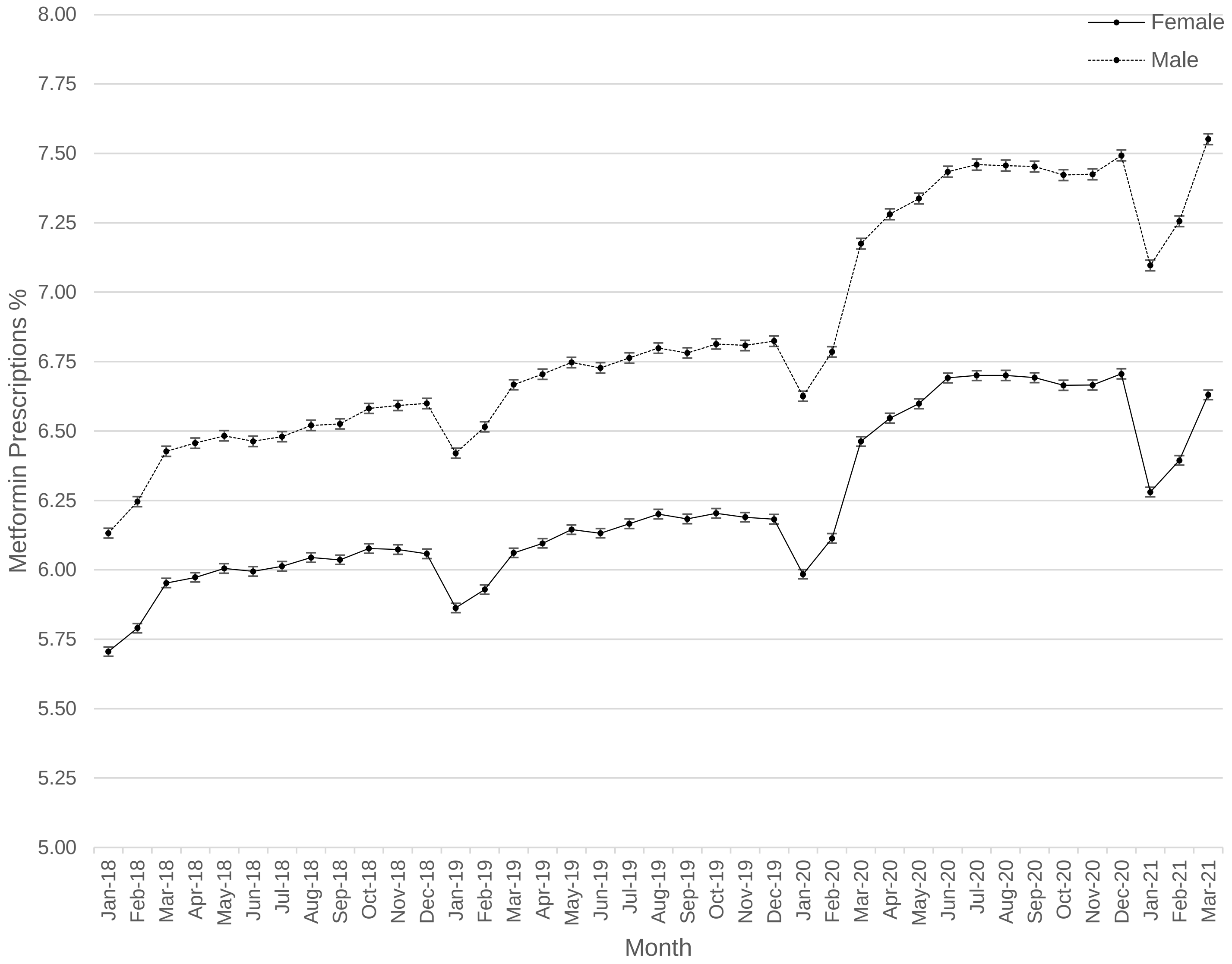

© 2021 Milani SA et al. *JAMA Network Open.*

eFigure 5. Monthly Rates of Metformin Prescriptions Among Adults Aged ≥18 in the US, 2018-2021, by Age

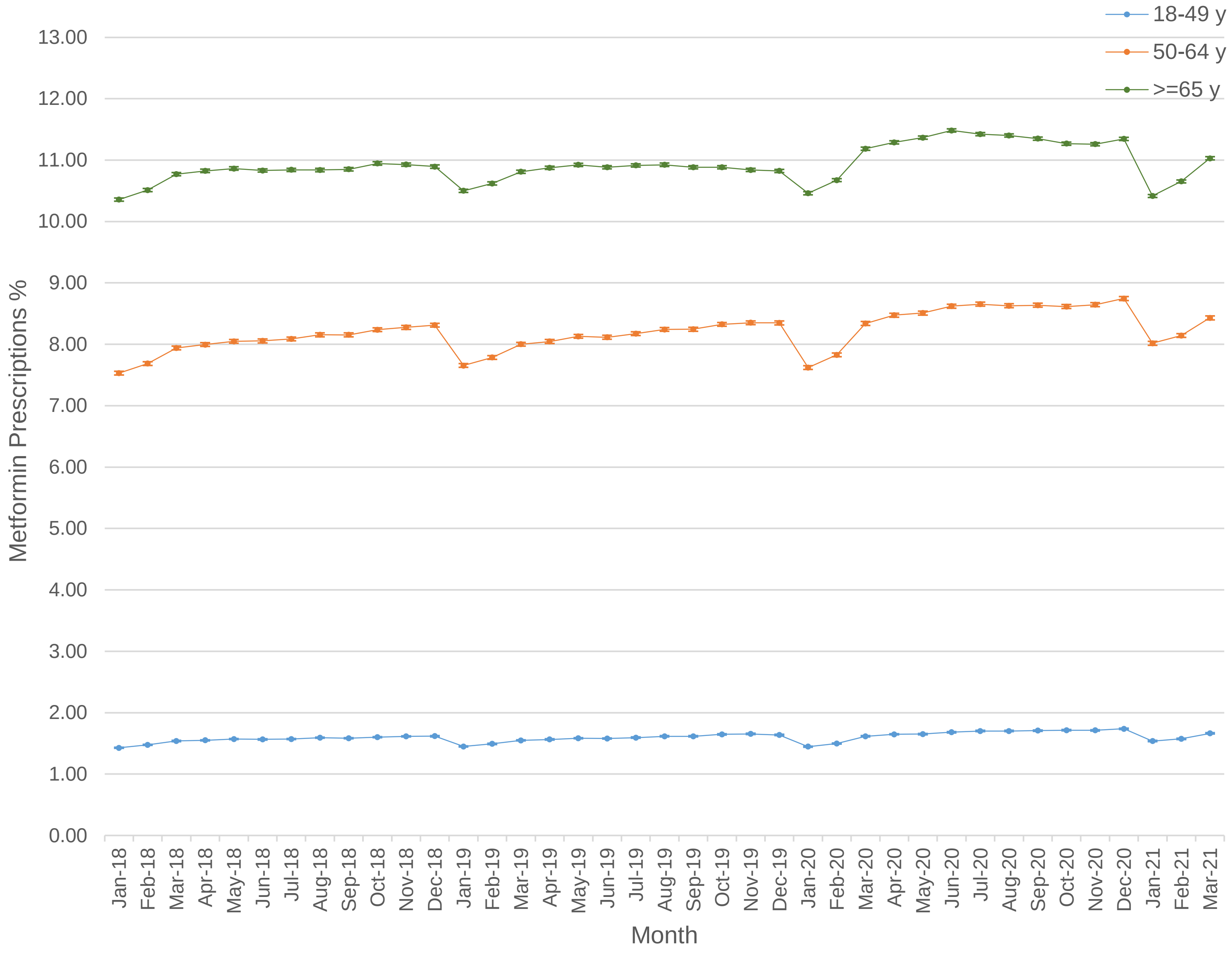

© 2021 Milani SA et al. *JAMA Network Open.*
